# Medical Society of the County of Albany

**Published:** 1871-03

**Authors:** James S. Bailey


ART. II.—*Medical Society of the County of Albany. Semi-Monthly Meeting, February 28th,* 1871.

REPORTED BY JAMES S. BAILEY, M. D.

Dr. Wm. H. Bailey, President, in the chair.

*Remarkable case of Hysteria.*—Dr. R. H. Sabin reported the following interesting case. Miss H., aged 21, of nervo-bilious temperament, when 14 years old, had a severe and protracted attack of typhoid fever, which left her debilitated for a long time. A few months after recovery she complained of pain in the right side and region of the liver. The attending physician diagnosed an abcess, and proposed to incise it, which was refused, and another physician was called. In the Fall of 1866, her uncle, Mr. B., visited her at her home in Ohio, and found her under the care of a Thomsonian. Not being satisfied with her treatment he brought her home with him, and called Dr. Jones, a Homeopathic of this city, who treated her about two years. He pronounced it a case of womb disease. As she did not improve under his care, Dr. Bonticow, of Troy, was called, who treated her for several months for an affection of the womb. Dr. Seymour, of Troy, saw the case in consultation with him, and concurred with him in this opinion. Dr. McChesny, of Troy, was next employed, who treated her for twelve months. She seemed to gradually improve under his treatment, which was entirely tonic and supporting. Before Dr. McChesny saw her, she had for twelve months maintained the sitting posture in bed night and day. The doctor, after a time, succeeded in enabling her to assume the horizontal position. Her left leg was drawn up, the heel pressing firmly against the vulva. No manipulation could dislodge it except by etherization. Then it could be straightened, but would return to this position as soon as consciousness returned. Several kinds of splints were used for the purpose of keeping the limb extended, but were unsuccessful.

During Dr. McChesny's service Dr. Sabin was called, in haste, to see her, as the nurse had by mistake administered a teaspoonful of tincture of iodine instead of tincture of valerian. Dr. Sabin made several visits with Dr. McChesney, who continued to treat the case with gradual improvement. The family becoming impatient, thinking convalescence too slow, Dr. Newton, an advertising quack, was next called, and by his mesmeric influence, or from some other cause, she was enabled to walk for a time. She then placed herself under a woman, a clairvoyant, living in Waterford, a few miles distant from her uncle's residence, whom she thought gave her some relief. Hearing of Dr. Peaslee, who was then lecturing in the Albany Medical College, she desired Dr. Sabin to accompany her to see him. On the 28th of September she intended to present herself at his clinique for an examination, but upon reaching the city she became so nervous she was conveyed to the city hospital, where Dr. Peaslee made an examination and found retro-flexion uteri, and thought all of her nervous symptoms came from that source, and that if the womb was restored to its normal position she would get well. On the following day he proceeded to replace it, after which he introduced a ring pessary in order to maintain it in position, and gave her hydrat chloral internally, and belladonna and morphia suppositories to allay irritation.

For the next six weeks, while every effort was made to keep the womb in position, she had the most violent spasms. When in them she assumed all the different positions that the imagination could conceive. Dr. Sabin, finding it impossible to control the spasms, removed the pessary. There being so much pain in the region of the left ovary, Dr. Sabin suspected an abscess forming, and applied a succession of blisters.

Dr. Newton was again consulted, and when called passed into a clairvoyant state, confirmed Dr. Sabin's suspicions, and said he could not do any thing more for her, but that if she lived long enough the matter would find its way out. Her spasms were described as being fearful, by Drs. Sabin and McChesny. She would lay upon her back, and her face would be turned over to the left shoulder; one hand would remain clenched tightly for weeks at a time. Dr. Sabin made an effort to straighten the left leg, and employed Day's inclined plain for that purpose; but the spasms came on before the operation was completed, and he applied the bandage to the limb one-fourth flexed, but the contractions were sufficient to burst the bandages, and he failed.

For the past two weeks, during the paroxysms, the heart and respiration seemed somewhat affected. She would cease occasionally to breathe for intervals of five minutes. Laterly, she had complained much of headache, and described the pain as a terrible distress and torture. In the treatment of this case, Dr. Sabin (as had all the physicians before him) had employed all of the known antispasmodics, nervines and anodynes of the pharmacopea, for her relief, without success; and, upon some occasions, had given more than an ounce of hydrat chloral in the course of twenty-four hours. Her bowels had usually been regular, but recently were somewhat constipated. Her appetite was usually craving; she had a great fancy for pickles; but recently her appetite was not so good. She menstruated regularly but somewhat scantily, and had some leucorrhœa.

*Autopsy.*—At the *post mortem* the attending physicinas were present, besides several physicians from Albany and Troy.

*External Appearances.*—The body was well nourished; the left leg somewhat atrophied.

*Abdomen.*—The womb presented the natural appearances of the virgin uterus; bladder healthy; kidney normal, as well as the rest of the abdominal viscera.

*Chest.*—The viscera seemed healthy in every portion of the chest.

*Head.*—The brain seemed healthy and natural in consistence. The madulla oblongata was free from disease, as well as the upper end of the spinal cord. Dr. Edward R. Hun examined the anterior crural nerve under the microscope, and found it healthy.

*Necrosis of Inferior Maxillary.*—Dr. JAMES S. BAILEY presented a section of three-quarters of an inch of the inferior maxillary, which was exfoliated. The patient was a little German girl aged five years. In September she was attacked with whoopingcough of an aggravated form, and her life was severely threatened. In December Dr. Bailey was consulted. She was much attenuated; and one inch from the symphysis, on the right side of the jaw, there was an ulcer which presented the appearance of an ulcer in the stomach, the edges were as smooth and even as if they had been cut by a punch; from this opening was discharging thin and unhealthy pus; the denuded necrosed bone was visible. Dr. Bailey insisted upon giving her a generous fluid diet, and by means of mild tonics she rapidly grew in strength and flesh. An operation was proposed for the removal of the necrosed portion, but the parents strenuously resisted. In February the father importuned

Dr. B. to operate; but as nature was acting her part so nobly, it was thought best to wait. Upon the 26th of February, Dr. B., by means of a strong pair of forceps, removed the portions exhibited from the symphysis back. It embraces the right central and lateral incisors, the canine and molar teeth. The specimen is remarkable on account of its showing upon one side a sack containing the dental pulp preparatory for the formation of a permanent tooth, and upon the other side is a well-formed permanent tooth making its way through. The doctor was unable to account for the cause of this necrosis, and was not able to give information in reference to the child's treatment previous to the consultation with himself.

Dr. C. A. Robertson offered the following resolutions, which were adopted:

*Whereas*, At a meeting of the organization, known as the "Homœopathic State Medical Society," held in this city, a series of resolutions was passed on the 15th day of February, instant, in which H. Van Aernam, M. D., United States Commissioner of the Bureau of Pensions, was severely reprobated for removing a homoeopathic practitioner from the office of Examining Pension Surgeon, because the theories professed by said practitioner respecting disease and treatment of it, were antagonistic to the conviction and well-grounded practice of the Medical Profession of the civilized world: and

*Whereas*, A passionate appeal to the community was contained in the utterances of said resolutions, made for the apparent purpose of arousing excitement and stimulating impulsive action against said Commissioner, by improperly representing his consistent and straight-forward conduct to be "an act of proscription for opinion's sake:" and

*Whereas*, A demand, couched in arrogant and extravagant language, was addressed to the Chief Magistrate of the nation, calling for the quick removal of a valuable public officer, deserving great praise for numerous improvements in his Bureau, because, in the conscientious discharge of his duty, he had unavoidably discriminated against one of a "class of practitioners," as they term themselves in said resolutions: and

*Whereas*, Delegates were appointed to visit the city of Washington, for the purpose of exercising an influence in behalf of said "class of practitioners," but prejudicial to the real and important interests of the Pension Bureau: *Therefore*,

*Resolved*, That this County Medical Society, which numbers about as many members as the entire "Homoeopathic State Medical Society," deems it proper to give formal expression of opinion concerning the matters involved in said resolutions.

*Resolved*, That Commissioner Van Aernam is entitled to commendation from the Medical Profession everywhere, and that this society thanks him for meeting and checking with promptitude and decision the encroachment of "any class of practitioners" who put themselves in an attitude of hostility to legitimate medicine.

*Resolved*, That, inasmuch as homoeopathic and other irregular practitioners have no *status* in the Medical Profession, Commissioner Van Aernam, would, as a physician, have contravened the spirit and insructions of the Profession, both of this state and of this country, had he continued in official position in his Bureau, any professed medical gentleman, with whom neither the Commissioner himself, nor any qualified Pension Surgeon could legitimately hold professional consultation.

*Resolved*, That the Medical Profession would regard with concern and earnest protest any disposition manifested on the part of the civil authorities of the nation to interfere with its deliberate judgment and condemnation of the dogmas of a "class of practitioners," who do not even profess to base their notions or practice on deductions from any recognized laws of physical science, and therefore cannot properly be termed physicians.

*Resolved*, That any reprehension by the government of Commissioner Van Aernam, in response to the demands of homoeopathic practitioners, that he be punished for the offences which they allege, would, in the opinion of this Society, be deemed by the Medical Profession at large, an unusual and unjustifiable interference in affairs pertaining exclusively to a great and dignified profession, done in behalf of a comparatively small "class of practitioners," who have attempted to perpetuate conjectural theories by the machinery of separate organization, and to secure notoriety by noisy clamors, like other transient, fanciful sects in the history of medicine; and would establish a precedent of a remarkable character, even in a monarchy, while here it would be unparalleled for inconsistency with republican ideas.

*Resolved*, That a copy of these resolutions be presented to his Excellency, the President of the United States; to the Honorable the Secretary of the Interior; to the Honorable Senators and Representatives in Congress from the State of New York; to the Surgeon General of the United States; and to the County and other Medical Societies of the State.

*Resolved*, That the Senators and Representatives from the State of New Yo k be requested to submit these resolutions to the attention of both Houses of Congress.

*Resolved*, That a copy of these resolutions be published in the newspapers of this city.

WM. H. BAILEY, President.

CHAS. H. PORTER, Secretary.

